# Severe *Staphylococcus aureus* infections in children: Case reports and management of positive Panton-Valentine leucocidin cases

**DOI:** 10.3389/fped.2022.1003708

**Published:** 2022-10-13

**Authors:** Sophie Goemanne, Anne Tilmanne, Dominique Biarent, Pierre Smeesters, Paolo Simoni, Bhavna Ansuya Mahadeb, Alfredo Vicinanza

**Affiliations:** ^1^Department of Pediatrics, Hôpital Universitaire des Enfants Reine Fabiola, Université Libre de Bruxelles (ULB), Brussels, Belgium; ^2^Division of Pediatric Infectious Diseases and Infection Prevention and Control, Hôpital Universitaire des Enfants Reine Fabiola, Université Libre de Bruxelles (ULB), Brussels, Belgium; ^3^Pediatric Intensive Care Unit, Hôpital Universitaire des Enfants Reine Fabiola, Université Libre de Bruxelles (ULB), Brussels, Belgium; ^4^Laboratory of Molecular Bacteriology, Université Libre de Bruxelles (ULB), Brussels, Belgium; ^5^Department of Radiology, Hôpital Universitaire des Enfants Reine Fabiola, Université Libre de Bruxelles (ULB), Brussels, Belgium; ^6^Department of Microbiology, Hôpital Universitaire des Enfants Reine Fabiola, Université Libre de Bruxelles (ULB), Brussels, Belgium

**Keywords:** *Staphylococcus aureus*, Panton-Valentine leucocidin, severe infections, children, methicillin-sensitive *Staphylococcus aureus*

## Abstract

**Background:**

*Staphylococcus aureus* is a well-known bacterium associated with carriage and responsible for different types of infections. The Panton-Valentine leucocidin (*PVL*) is a key virulence factor causing tissue necrosis. *PVL* can, however, be present in both benign and life-threatening infections.

**Case reports and management:**

We present three pediatric severe infections occurring over a period of only three weeks, in February 2021, and caused by genetically unrelated methicillin-sensitive *Staphylococcus aureus* producing *PVL* in a tertiary children’s hospital in Belgium. The first one presented with necrotizing pneumonia, the second one with a neck abscess extended to the mediastinum, and the last one had sacral osteomyelitis complicated by endocarditis. The management of these infections is mostly based on expert opinions. The most appropriate treatment seems to be the combination of early surgical drainage of infected collections with an antibiotic regimen associating two antibiotics; beta-lactams and either clindamycin or linezolid. Human immunoglobulins also appear to be useful as adjunctive therapy.

**Conclusion:**

*PVL*-producing *Staphylococcus aureus* is associated with life-threatening infections in children. Prompt management is needed including surgery and appropriate antibiotic regimens.

## Introduction

*Staphylococcus aureus (SA)* is one of the most frequently isolated bacteria in hospital-acquired and community-based infections. It is also frequently recovered from asymptomatic children with carriage rates ranging from 10 to 57% (1).

*SA* carries several virulence factors (about 50) that help to circumvent the human immune system (2). Panton-Valentine leucocidin (*PVL*) is an exotoxin allowing the *SA* to form pores in some host cells (neutrophils, monocytes, and macrophages), leading to tissue necrosis (3). Although *PVL* was first described by Van de Velde in 1894 (4), Panton and Valentine described the association between the toxin and skin and soft tissue infections in 1932 (4, 5). The presence of the *PVL* gene in *SA* strains varies in different parts of the world and the prevalence is much higher in strains from Africa and Latin America than in European ones (6, 7). Even if its prevalence in Europe has not been well-established, some authors have rated it at less than 5% (4, 5). This gene can be carried by both methicillin-sensitive and methicillin-resistant *SA* (*MSSA* and *MRSA*, respectively). Nowadays, various types of infections can be caused by both *MSSA* and *MRSA* carrying *PVL (SA-PVL* +), the most frequent ones remaining skin and soft tissue infections that are usually complicated by multiple lesions and deep-seated abscesses and because of their recurring episodes. Nevertheless, severe and life-threatening infections might develop even in young and immunocompetent patients (3, 8–10).

Many series of severe *SA-PVL* + infections have been reported in the pediatric population all over the world (10–12), underlining the interest in rapid *PVL* detection in the case of severely *SA*-infected patients (13). However, there is probably a detection bias for these infections, as *PVL* is not systematically requested after an *SA* isolation.

The purpose of our work is to describe three consecutive pediatric cases of severe *MSSA-PVL* + infections in light of the recent literature. This study was carried out with the agreement of the ethics committee of our institution (CEH n°73/21).

## Case description

### Case 1

A 22-month-old African boy, born at full-term after normal pregnancy in Belgium, with no relevant medical history nor recent travel history and fully vaccinated according to the Belgian schedule, presented with dry cough and high fever for 3 days and respiratory distress for 24 h.

At admission, the child appeared grumpy, had tachycardia, and had a high fever (40°C). The first blood test showed mild inflammatory syndrome.

Pneumonia with pleural effusion was diagnosed clinically and on a chest X-ray. Intravenous (IV) penicillin (500,000 units/kg/day) was the first chosen antibiotic. Respiratory conditions worsened quickly, requiring a transfer to our pediatric intensive care unit (PICU) 6 h after admission.

Then, another chest X-ray showed a massive pleural effusion with tracheal deviation and a partial collapse of the left lung. At that time, the inflammatory syndrome had increased showing a discrepancy between leukocytes count (4160/μl) and C-reactive protein (CRP; 265 mg/L). IV flucloxacillin (200 mg/kg/day) and clindamycin (30 mg/kg/day) immediately replaced penicillin. The pleural effusion required a surgical drain placed by video-thoracoscopy.

Two days after thoracoscopy, the patient’s general status deteriorated and he developed a septic shock requiring invasive ventilation, fluid resuscitation, and vasoactive amines. The shock remained refractory despite the adjunction of hydrocortisone, and an acute respiratory distress syndrome (ARDS) arose. Impaired cardiac function and ARDS led to venoarterial extracorporeal membrane oxygenation (ECMO) support for 9 days whereas invasive ventilation was required for 6 weeks. Acute renal insufficiency (KDIGO stage 3) was treated by continuous hemodiafiltration for 5 weeks with complete resolution of the renal insufficiency before discharge.

Because of the brutal degradation, the antibiotic therapy was transiently extended to IV ceftriaxone (100 mg/kg/day) and IV vancomycin (40 mg/kg/day continuously after a charge IV dose of 10 mg/kg) for 2 days, until obtaining the isolation of an *MSSA* from the pleural sample. Blood cultures remained negative. Flucloxacillin was finally administrated for 6 weeks and clindamycin for 10 days. IV human immunoglobulins (2 g/kg) were administered due to the initial suspicion of toxic shock syndrome.

**FIGURE 1:**
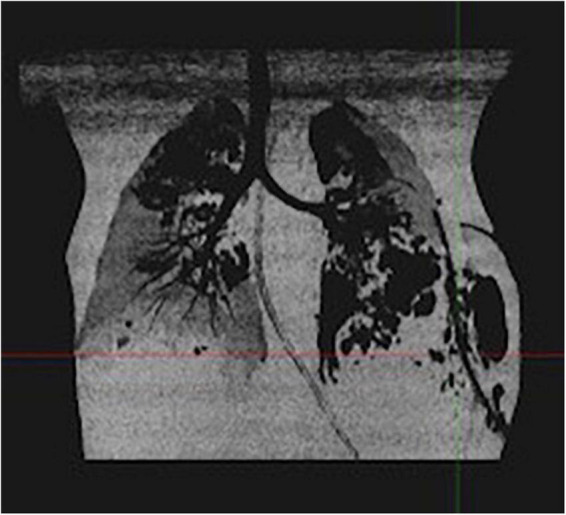
Thoracic CT scan of case 1: bilateral pneumatoceles and necrotizing pneumonia.

Gradually, pulmonary lesions turned into bilateral pneumatoceles and necrotizing pneumonia (Figure 1). The pleural drain was left in place for several days because of persistent pneumothorax secondary to bronchopleural fistulas.

Based on the severe presentation and the unusual aggressive clinical course, we requested both *spa*-typing and the detection of a *PVL* toxin by polymerase chain reaction (PCR) on the *SA* recovered from the pleural sample and results came positive 13 days after admission (*spa*-type t021). Other exotoxins (toxic shock syndrome toxin-1 – *TSST-1*, exfoliatin A – *eta*, and exfoliatin B – *etb*) were negative. Enterotoxins had not been tested. The presence of *PVL*, *TSST-1*, *eta*, and *etb* genes was tested by end-point PCR using primers previously described (14). No contact with people having skin or soft tissue infections have been reported.

The patient showed mild short-term neurological disability (mild encephalopathy on the electroencephalogram) but gradually improved. One month after his admission, a cerebral magnetic resonance imaging (MRI) showed intraventricular, right lateral ventricle occipital horn, and right frontal white matter hemosiderin deposits, as well as ventricular dilatation. On a control 5 months later, the hemosiderin deposits remained with a significant decrease of ventricular dilatation.

At the 2-month follow-up, he presented unsteady walking, poor language for his age, and also sleep disorders. He recovered stable walking and good sleep quality at the 9-month follow-up even if his language remained underdeveloped for age.

The pneumatoceles were still seen on chest X-ray at the 2-month follow-up but without clinical impact. Chest X-ray significantly improved 1 year later.

### Case 2

A 21-month-old African girl born in Belgium without medical history or travel history (for her and her family) and fully vaccinated, presented with a 3-day high fever to another emergency department where treatment by oral amoxicillin had been initiated. Four days later, because of persistent high fever and the onset of a one-sided cervical bulk, she came to the emergency room of our institution.

**FIGURE 2:**
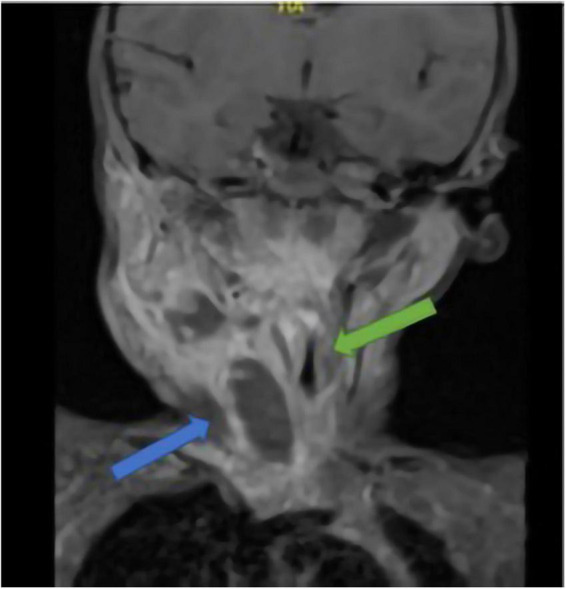
Cervical MRI of case 2; blue arrow: abscess; green arrow: trachea stenosis.

Cervical bulk was indurated, painful, and measured 3 cm on its long axis, at palpation. Initial blood analysis demonstrated a major inflammatory syndrome with a white blood cell count of 22,330/μl (with 16,190/μl absolute neutrophils) and a CRP of 180 mg/L. Indeed, a cervical MRI showed an abscess cavity measuring 10 cm long and 2 cm wide on a necrotic lymph node. This abscess extended to the anterior parapharyngeal space fusing to the middle mediastinum, next to cardiac vessels. Its massive extension reduced the size of the trachea (Figure 2).

After diagnostic imaging, IV ceftriaxone (100 mg/kg/day) associated with IV clindamycin (30 mg/kg/day) were started and the abscess was drained under ultrasound guidance. *MSSA* was isolated from the abscess sample but blood cultures stayed negative. After microbiological results, antibiotic therapy was changed to flucloxacillin (200 mg/kg/day) only and continued for 15 days (10 intravenously). A *PVL* test yielded positive in a *spa*-type t008 isolate. The PCR for other exotoxins (*TSST-1*, *eta*, and *etb*) were negative. Enterotoxins had not been investigated. She did not report any close contact with people having skin or soft tissue infections.

The total length of her hospital stay was 11 days including 5 at PICU. She had no sequelae of the infection at the 1-year follow-up.

### Case 3

A 14-year-old fully vaccinated African girl born in Belgium, without any medical background and whose last travel with her family had been 10 years before to Congo Brazzaville. She was admitted to the Emergency Department of our institution for chest pain and dyspnea associated with deterioration of general condition for a few hours. She also had fever (around 38.7°C) and cough for 3 days.

A week before these complaints, she had a dizzy spell in the shower, which resulted in a fall. The day after she started to complain of coccygeal pain.

She presented with pulmonary infection and a major inflammatory syndrome on a blood test (CRP at 275 mg/L). She was admitted to the PICU for circulatory failure needing hemodynamic support with norepinephrine and milrinone for 2 days. The differential diagnosis between toxic shock syndrome, pediatric inflammatory multisystemic syndrome, and septic shock with pulmonary onset led to the initiation of IV broad-spectrum antibiotic therapy, ceftriaxone (100 mg/kg/day), and clindamycin (900 mg 3 times per day) combined with IV human immunoglobulins (2 g/kg).

**FIGURE 3:**
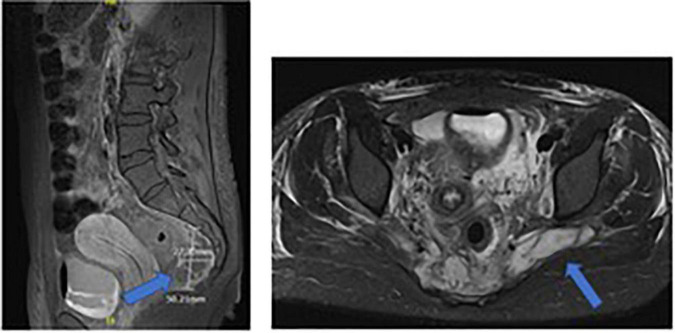
Sacral MRI of case 3: sacral osteomyelitis and multiple adjacent abscesses.

At admission to PICU, a pancarditis with endocarditis of the mitral valve apparatus and pericardial effusion were highlighted. The extended workup showed cerebral, pulmonary, renal, and cutaneous emboli. An MRI of the pelvis, performed because of the history of coccygeal pain, revealed sacral osteomyelitis and multiple adjacent abscesses (Figure 3). Therefore, the suspected mechanism of septic shock was endocarditis with multiple emboli secondary to sacral osteomyelitis.

*MSSA* was found in multiple samples, namely: blood cultures (during 7 days), urine cultures, and cultures from pericardial fluid and pelvic abscesses. The liquid from ascites was negative.

From the first identification of *MSSA*, the second day of hospitalization, antibiotic treatment was changed for high-dose IV flucloxacillin (200 mg/kg/day) and clindamycin, held for 8 weeks and 11 days, respectively.

A test for *PVL* was found to be positive in a *spa*-type t5691 isolate. Investigations for other exotoxins (*TSST-1*, *eta*, and *etb*) were negative. Enterotoxins had not been tested. She had never been in contact with anyone presenting with recurrent skin or soft tissue infections.

Sustained bacteremia for 7 days despite a well-conducted antibiotic therapy and the numerous infected sites secondary to emboli required the drainage of the infected site (pelvic abscesses) to stop blood cultures to be positive.

Seven months after the beginning of this medical history, the patient started sport again with good tolerance. She had no sequelae.

## Discussion

We reported three consecutive, but genetically unrelated, severe pediatric cases of *PVL* + *MSSA* infections in our PICU. Due to their severe clinic presentations associated with their very close admissions in PICU (over a 3-week time only) and their African origins, a cluster of *MSSA-PVL* + infections was suspected in our three patients, and a *PVL* was requested for each of them. However, typing revealed three unrelated strains (*spa*-type t021, *spa*-type t008, and *spa*-type t5691, for cases 1, 2, and 3, respectively). This could suggest the hypothesis that *PVL* may remain not sufficiently explored and, therefore, *SA-PVL* + infections underdiagnosed.

Several clones of *SA-PVL* + emerged in different parts of the world. The most famous is USA300 (belonging to strain ST8), an *MRSA* strain, widespread in the USA. In this country, the majority of *PVL* strains are *MRSA*, whereas in Europe, the majority are *MSSA* (3, 15–17).

In 2020, in Belgium, 451 community-acquired isolated *SA*, including 201 *MRSA* and 250 *MSSA*, were identified by the National Reference Center (NRC) (18). The proportion of *PVL* + isolates was 47 and 21.6% among *MRSA* and *MSSA*, respectively (18). Of note, *PVL* investigation is only undertaken if required by a clinician.

Each practitioner should be aware of this issue, might request a *PVL* detection if clinically suspected, and quickly implement the appropriate treatment before the microbiological confirmation.

The well-known risk factors for the development of severe diseases by *SA-PVL* + are close contact with people having purulent, recurrent, deep-seated, and multi-site skin or soft tissue infections (19), as well as travel history to high prevalence regions, such as Africa and Latin America, especially for patients born and living in low prevalence regions such as Europe (6, 7, 20). That was not the case for any of our patients. Initial leukopenia (with a discrepancy between white blood cells and CRP), like in case 1, and signs of hemorrhage of the airways might be suggestive of *PVL* (9, 21, 22). The clinical presentation can be immediately severe and very acute with fast degradation, like in cases 1 and 3. Necrosis, abscesses, and destruction of tissues are due to the cytotoxic effect of *PVL* (21). As in our case 1, staphylococcal necrotizing pneumonia should be considered in the presence of multilobar lung infection with possible pneumatoceles. If a *SA* produces *PVL*, there may be a rapid progression toward septic shock and ARDS (9, 21). The morbi-mortality is higher in necrotizing pneumonia due to *SA* if it produces *PVL* (9) with a mortality rate above 50% in certain series (22).

However, a systematic review by Vardakas et al. (23) comparing the outcomes of pneumonia due to *MSSA* and *MRSA*, both of which producing *PVL*, showed no difference in mortality rates between patients with *MRSA-PVL* + pneumonia (*n* = 76) and patients with *MSSA-PVL* + one (*n* = 31). Likewise, another study by Sicot et al. (24) showed no difference in mortality rate in pneumonia caused by either *MSSA-PVL* + or *MRSA-PVL* +.

ECMO support appears to be helpful in the management of ARDS in *SA-PVL* +-necrotizing pneumonia with or without hemodynamic instability (25). Like our case 1, two other pediatric patients in the USA, requiring venoarterial ECMO, were discharged alive with good clinical outcomes (25). Other case reports of two pediatric patients and two adult cases (26) have demonstrated the efficacy of ECMO for refractory hypoxemia in *SA-PVL* + induced-ARDS.

Some cases of bone and joint infections due to *PVL* show signs of sepsis, multi-site infections (metastatic abscesses), and local extension (subperiosteal abscesses and/or soft tissue expansion) despite appropriate treatment (10, 21, 27–30). We were able to find all these signs in the clinical presentation of case 3. We found only one other case of osteomyelitis complicated by endocarditis, in a case series of 11 children with severe invasive infections caused by *MSSA-PVL* +, in the UK, although he had neurological sequelae (10), unlike our patient.

Due to a lack of comparative studies, the management of infections by *SA-PVL* + is primarily based on expert opinions. Gillet et al. published a review of treatment options for different types of infections caused by *SA-PVL* + (21). The Health Protection Agency in the UK also published in 2008 a Guidance on the diagnosis and management of *SA-PVL* + infections (31). The ideal treatment of those infections should combine surgical drainage of infected sites, inhibition of the *PVL* production (with appropriate antibiotics), and blocking the toxic effects of *PVL* after its production (4, 21, 27, 31). The choice of antibiotics is therefore one of the keys. The minimum inhibitory concentration can be difficult to achieve in necrotic tissues (4). Some toxins, like *PVL*, are produced during the stationary phase of the bacterium. Beta-lactams (like oxacillin), antibiotics usually used against *SA*, are not very effective during this phase. Second, beta-lactams, at the sub-inhibitory concentration (as in necrotic tissues), could increase *PVL* production (4). An *in vitro* study by Dumitrescu et al. (32) showed a decrease in *PVL* production when a beta-lactam is combined with an antibiotic active on protein synthesis, in particular with clindamycin but also with linezolid. This effect should be present even if the antibiotic concentration on infected sites is far from the minimum inhibitory concentration.

Therefore, the most effective treatment against *SA-PVL* + seems to be the combination of a beta-lactam (like flucloxacillin at high doses – 200 mg/kg/day) with a protein synthesis inhibiting antibiotic (such as clindamycin at 30 mg/kg/day or linezolid at the same doses) (21, 31).

Another treatment option against this bacterium is the use of human immunoglobulins acting on the toxic effects of *PVL*. Another *in vitro* study by Gauduchon et al. showed that human immunoglobulins neutralize pore formation and the cytotoxic effect of *PVL* (33). Although there are no *in vivo* studies, several case reports showed improvement after the use of immunoglobulins in patients with severe *SA-PVL* + infections (4, 10, 22, 34). In line with the literature, two of our patients (case 1 and case 3), with the most severe presentations, received immunoglobulins as adjuvant treatment, which may have contributed to their clinical improvement. Two studies, one involving pediatric series (35) and one both pediatric and adult patients (36), demonstrated that patients with *SA-PVL* + infections can develop specific IgG against *SA-PVL* +.

## Conclusion

*Staphylococcus aureus* presents a lot of virulence factors including the production of toxins like Panton-Valentine leucocidin.

We highlighted that *MSSA*-producing *PVL* could lead to various severe and life-threatening infections that might involve young and immunocompetent patients.

This case series may help to increase awareness of attending physicians to reduce the rate of overlooked *SA-PVL* + infections, especially in regions with low *PVL* prevalence as in Europe.

The role of clinicians in recognizing these infections could be an important public health issue as initiating a prompt treatment, before receiving the confirmation of *PVL* production, might be life-saving.

## Data availability statement

The original contributions presented in this study are included in the article/supplementary material, further inquiries can be directed to the corresponding author.

## Ethics statement

This study was approved by the ethics committee of our institution “Comité d’Ethique de l’Hôpital Universitaire des Enfants Reine Fabiola, Brussels, Belgium (CEH n° 73/21)”. Written informed consent to participate in this study was provided by the participants’ legal guardian/next of kin.

## Author contributions

SG conceived and designed the work and wrote the manuscript. AV drafted, structured the manuscript, and revised it. AT, DB, and PSm revised the manuscript. All authors interpreted the data, managed the patient, contributed to the article, and approved the submitted version.

## Abbreviations

ARDS: acute respiratory distress syndrome
CRP: C-reactive protein
ECMO: extracorporeal membrane oxygenation
ETA: Exfoliatin A
ETB: Exfoliatin B
IV: intravenous
MRI: magnetic resonance imaging
MRSA: methicillin-resistant Staphylococcus aureus
MSSA: methicillin-sensitive Staphylococcus aureus
NRC: National Reference Center
PCR: polymerase chain reaction
PICU: pediatric intensive care unit
PVL: Panton-Valentine leucocidin
SA: Staphylococcus aureus
TSST-1: toxic shock syndrome toxin-1.
